# Insight into the invasion process and immune-protective evaluation of Tp0971, a membrane lipoprotein from *Treponema pallidum*

**DOI:** 10.1128/spectrum.00047-23

**Published:** 2023-10-19

**Authors:** Xiaohong Zhang, Junxia Duan, Yali Wang, Bibo Xie, Jie Zhou, Sisi Zhao, Weiguo Yin, Peng Liu, Feijun Zhao

**Affiliations:** 1 Institute of Pathogenic Biology and Key Laboratory of Special Pathogen Prevention and Control of Hunan Province, Department of Clinical Laboratory Medicine of the First Affiliated Hospital, Hengyang Medical College, University of South China, Hengyang, China; 2 Department of Clinical Medicine Undergraduate, Hengyang Medical College, University of South China, Hengyang, China; 3 Laboratory Department, Qingyuan People’s Hospital, Qingyuan, China; Shenzhen Bay Laboratory, Shenzhen City, China

**Keywords:** *Treponema pallidum*, Tp0971, invasion process, immune-protective effect

## Abstract

*Treponema pallidum* (*T. pallidum*), the pathogen of syphilis, can invade organisms through mucous membranes or broken skin and proliferate in the host. It spreads rapidly and causes chronic systemic multi-organ damage. Currently, the invasion and pathogenesis of *T. pallidum* remain a mystery. In this study, we established a *T. pallidum* infection model in New Zealand rabbits, and the *T. pallidum* burden in various tissues and organs was detected to investigate the dynamic spread of *T. pallidum* in different organs. Our results indicated that the *T. pallidum* burden in rabbits was in a cyclic and repeated dynamic process of decreasing and increasing after infection. In addition, the localization of lipoprotein Tp0971 was confirmed by using the gel microdroplet method. We found that Tp0971 might be a membrane lipoprotein that exists in the inner and outer membranes of *T. pallidum*. The immune-protective effect of the *T. pallidum* infection-dependent antigen Tp0971 was evaluated. Tp0971/CpG can induce high levels of Tp0971-specific antibodies, delay skin damage, and promote healing at the infected sites of *T. pallidum* in New Zealand rabbits. This indicated that Tp0971 may serve as a vaccine antigen candidate. Our results provide new ideas for future research on the proliferation, spread mechanism, and vaccine development of *T. pallidum*.

**IMPORTANCE:**

The past two decades have seen a worldwide resurgence in infections caused by *Treponema pallidum* (*T. pallidum*) subsp. *pallidum*, the syphilis spirochete. The well-recognized capacity of the syphilis spirochete for early dissemination and immune evasion has earned it the designation “the stealth pathogen.” There are many hurdles to studying syphilis pathogenesis, most notably the difficulty of culturing and genetically manipulating *T. pallidum*, as well as the absence of an effective vaccine for *T. pallidum* prevention. *T. pallidum* infection in humans is a complex and lengthy process. In this study, we investigated the invasion process and the function of the infection-dependent antigen Tp0971 as an immunogen to inhibit the dissemination of *T. pallidum* in an animal infection model. This enables a better understanding of the specific pathogenic mechanism of this pathogen, syphilis pathogenesis, and vaccine research.

## INTRODUCTION

*Treponema pallidum* (*T. pallidum*) subsp. *pallidum*, an invasive spirochete and the pathogen of syphilis, is responsible for a chronic and sustainable systemic disease. This pathogen is transmitted primarily through sexual contact, placental transmission, or blood transfusion (1, 2). Generally, *T. pallidum* invades the host organism through damaged skin tissues or mucosa (3, 4); at the early stage of infection, the proliferation of *T. pallidum* in mucosa or damaged skin tissues results in some clinical symptoms, such as hard chancre, lymphadenopathy, syphiloderm, and chronic inflammation. It widely spreads to other tissues and organs through blood circulation and lymphatic vessels (5 – 8). Patients without effective treatment undergo an asymptomatic incubation period after infection. However, *T. pallidum* continues to replicate and spread in the body. Twenty-five percent of untreated individuals will develop tertiary syphilis, which can manifest through many symptoms, such as serious skin or visceral lesions, bony involvement, and even destructive cardiovascular and nervous system damage (9, 10). However, the spread process and pathogenesis of *T. pallidum* remain unclear.

The outer membrane of *T. pallidum*, which serves as the ultrastructural basis for its well-recognized capacity for invasiveness, immune evasion, and persistence (2) and as the hard cultivatable agent of venereal syphilis, has long been the subject of misconceptions and controversy. The outer membrane is the barrier to antibody binding, it contains a paucity of integral membrane proteins, and the majority of the spirochete’s immunogenic lipoproteins are periplasmic (11). The outer membrane protein Tp92, the only *T. pallidum* outer membrane protein that has structural features similar to the outer membrane proteins of other Gram-negative bacteria, recognizes CD14 and Toll-like receptor 2 (TLR2), transfers the signal to a downstream pathway, and activates NF-κB to mediate the production of IL-8. This mechanism may help *T. pallidum* escape recognition and elimination by the host innate immune system (12). Tp0136, another outer membrane protein of *T. pallidum*, has the ability to bind the fibronectin of host cells, which plays a crucial role in the pathogenesis of syphilis and promotes the migration of HMEC-1 cells by inducing CCL2 expression via the interaction of the fibronectin RGD domain with integrin β1 and the CCL2/CCR2 signaling pathway, and these interactions may contribute to the mechanisms that increase the capacity for self-healing infection of syphilis (13). According to the study of Deka et al. and Brautigam et al., the Tp0971 gene product of *T. pallidum* (Tp0971) is a periplasmic lipoprotein that is believed to be anchored to the inner membrane of this organism (14, 15). Additionally, Tp0971 has the ability to bind lactoferrin and contains binding sites for Fe^2+^ and Cu^2+^. As a result, it is believed to play a significant role in metal ion homeostasis (14, 15). Metal ions like Fe^2+^ and Cu^2+^ have significant implications for the physiology and toxicity of treponema during its metabolism (16 – 19). In our previous animal experiments, we observed a high expression of antibodies against Tp0971 in live *T. pallidum*-infected rabbits, which is consistent with the characteristics of infection-dependent antigens (20). Many reports have demonstrated that infection-dependent antigens are crucial factors for pathogenic infections and provide new options for diagnostic markers or vaccine development (21 – 23). The cellular location of Tp0971 is still controversial.

The global incidence of syphilis has been on the rise, with approximately 12 million new syphilis cases occurring worldwide every year, according to the estimation by the World Health Organization. However, there is no effective vaccine to prevent and control syphilis (24). CpG oligodeoxynucleotides (CpG ODNs) are safe and effective immune adjuvants that activate B lymphocytes and plasmacytoid dendritic cells through the TLR9 pathway, inducing the secretion of IFN-γ and IL-6, activating the production of Th1-type cytokines, and inducing CD8^+^ cell-mediated immune responses. Immunization of New Zealand rabbits using CpG adjuvants in combination with Tp92 protein can induce a strong protective immune response (25, 26). Hence, we evaluated the localization and immune-protective effects of Tp0971 with CpG. At the same time, the spread of *T. pallidum* in New Zealand rabbits was estimated.

## RESULTS

### The spread of *T. pallidum* in New Zealand rabbits

**Fig 1:**
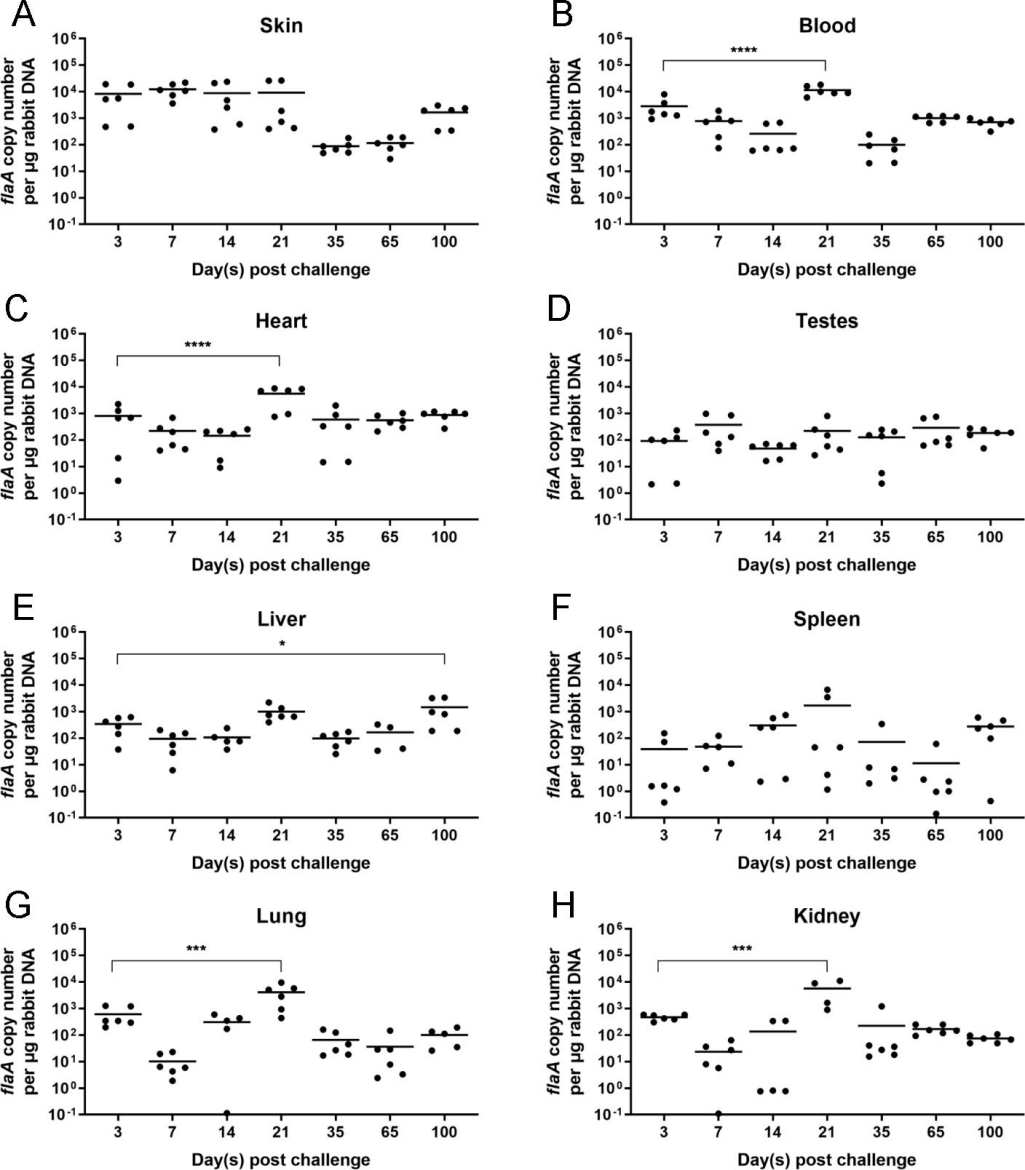
The *T. pallidum* burden in New Zealand rabbits’ tissues after infection. The *T. pallidum* burden was evaluated by qRT-PCR during the time course. (**A to H**) The DNA of the *flaA* gene in the skin, blood, heart, testes, liver, spleen, lung, and kidney was analyzed in panels A, B, C, D, E, F, G, and H, respectively. Statistical significance is indicated with asterisks (*P < 0.05; **P < 0.01; ***P < 0.001; ****P < 0.0001).

**Fig 2:**
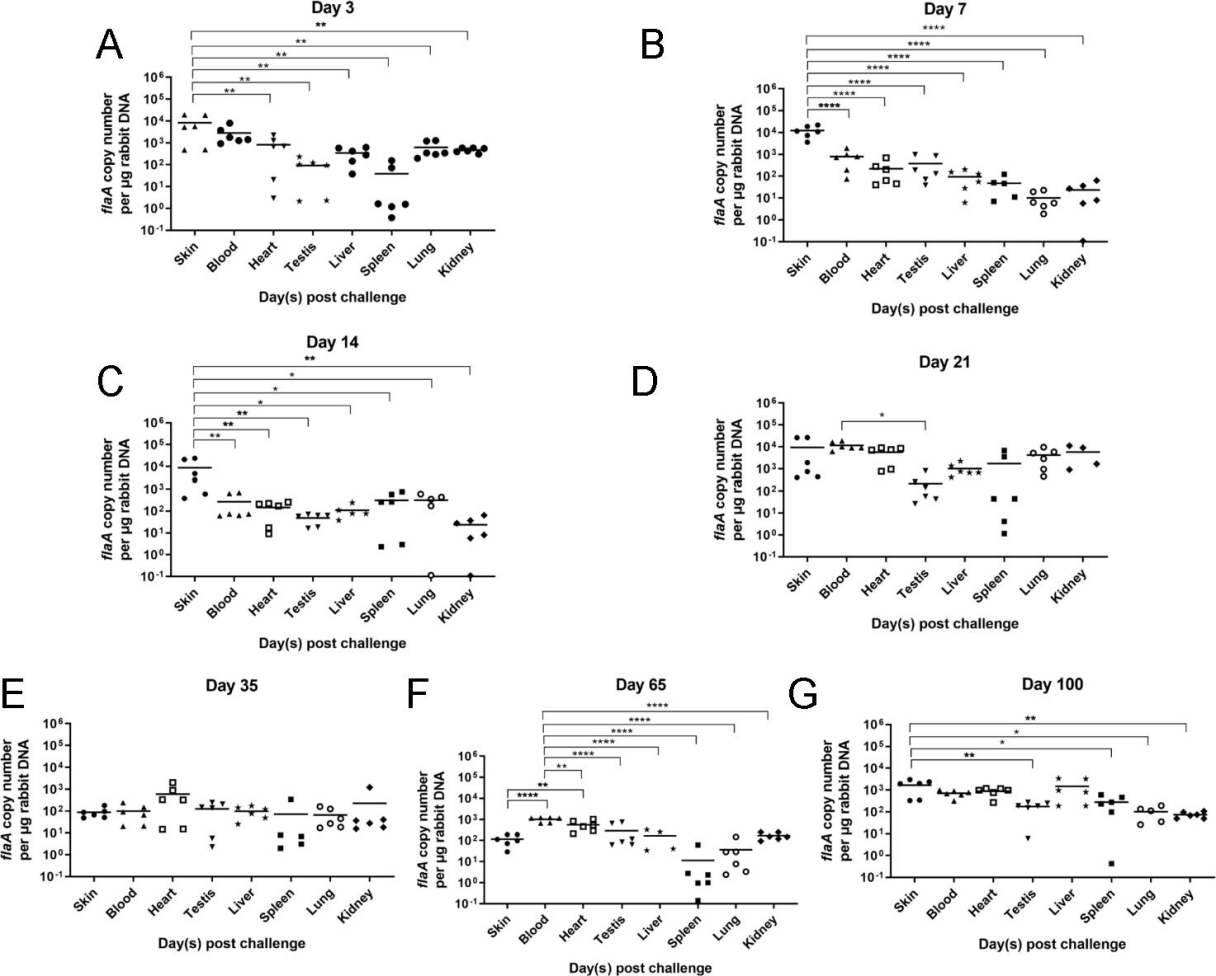
The *T. pallidum* burden in New Zealand rabbits presented at different infection times. The *flaA* DNA expression level of different New Zealand rabbits’ samples was detected by qRT-PCR. (**A to G**) The DNA expression level of the *flaA* gene on the 3rd, 7th, 14th, 21st, 35th, 65th, and 100th days after *T. pallidum* infection was determined in panels A, B, C, D, E, F, and G, respectively. Statistical significance is indicated with asterisks (*P < 0.05; **P < 0.01; ***P < 0.001; ****P < 0.0001).

We established a *T. pallidum* infection model in New Zealand rabbits for the purpose of *T. pallidum* invasion investigation. After infecting New Zealand rabbits with *T. pallidum*, we analyzed the spread trends of *T. pallidum* in the tissues of New Zealand rabbits using quantitative real-time PCR (qRT-PCR) at 3, 7, 14, 21, 35, 65, and 100 days. We found that *T. pallidum* was detectable in the skin, blood, heart, testes, liver, spleen, lung, and kidney on the third day after infection (Fig. 1). The *T. pallidum* burden in the blood, heart, lung, and kidney reached the most significant maximum on the 21st day (Fig. 1B, C, and H). However, the *T. pallidum* burden in the liver had a significant increasement until the 100th day of infection (Fig. 1E). The skin, testes, and spleen did not exhibit significant alterations in the *T. pallidum* burden (Fig. 1A, D, and F). Subsequently, we analyzed the *T. pallidum* burden in tissues and organs at different time points of infection. As shown in Fig. 2, however, *T. pallidum* was mainly enriched at the inoculation sites and spread to all tissues on the third day after infection (Fig. 2). However, the *T. pallidum* burden in the skin was significantly higher than that in the blood and other tissues (Fig. 2A). This difference was more obvious on the seventh day (Fig. 2B) but disappeared on the 21st and 35th days (Fig. 2D and E). Interestingly, the *T. pallidum* burden in the skin was significantly higher than that in the blood and heart, with no difference observed in other tissues. However, on the 65th day, the *T. pallidum* burden in blood was higher than that in other tissues with strong significance, but it disappeared again on the 100th day (Fig. 2F and G). However, on the 100th day, there are still some differences observed among the *T. pallidum* burden in the skin, testes, spleen, lung, and kidney (Fig. 2G). Therefore, the *T. pallidum* burden exhibits a dynamic trend at different stages of infection.

### The localization of Tp0971 determined by the gel microdroplet method

**Fig 3:**
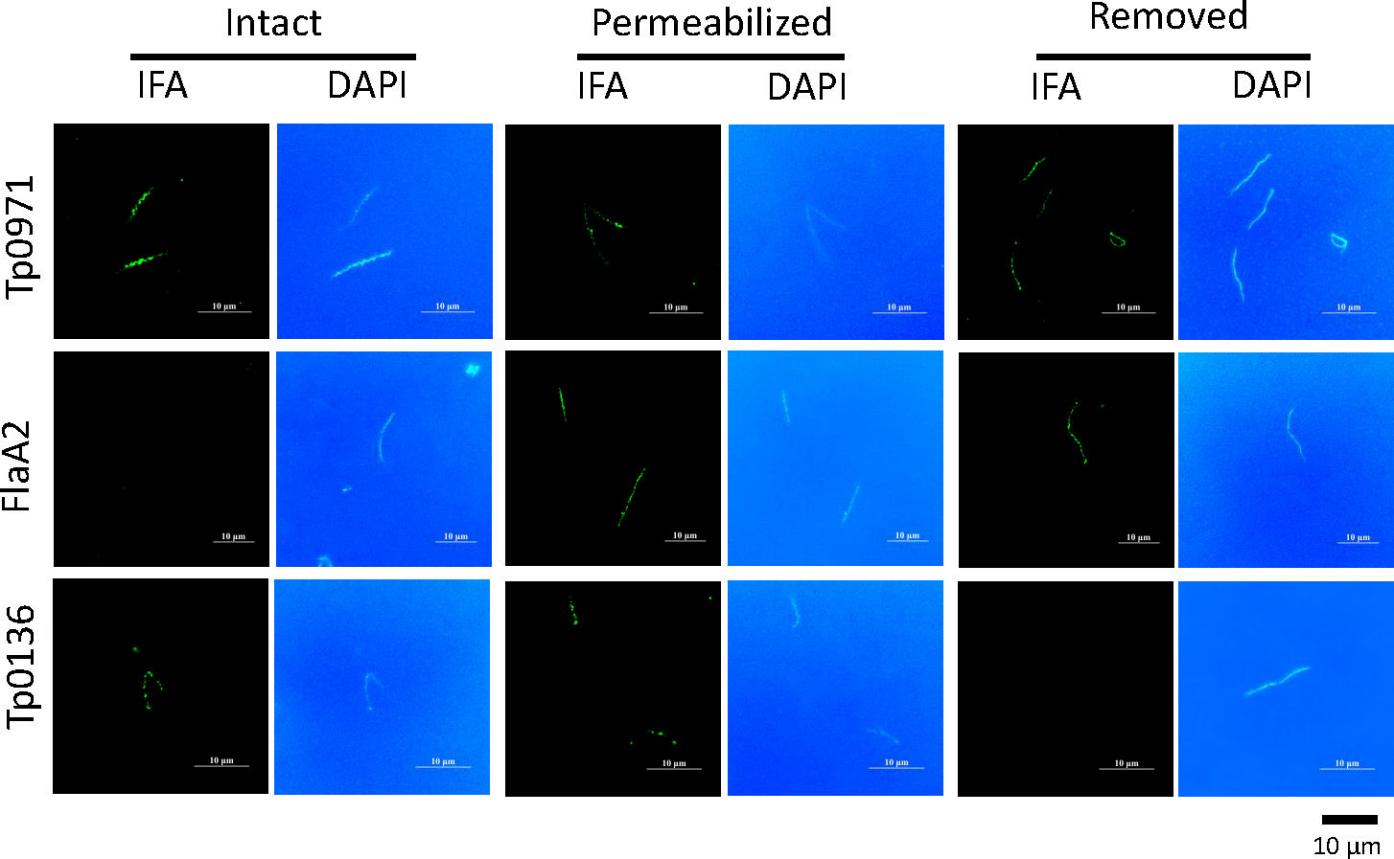
The localization of Tp0971 in *T. pallidum. T. pallidum* encapsulated in agarose gel was exposed to mouse antibodies anti-Tp0971, anti-FlaA2 (Tp0664), and anti-Tp0136. The *T. pallidum* with intact outer membrane group, the *T. pallidum* membrane permeated by Triton X-100 group, and the *T. pallidum* without outer membrane group were marked as the intact group, permeabilized group, and removed group, respectively.

The inner flagellum is the organelle responsible for the motility of *T. pallidum* and is located at the periplasm of the cell wall of *T. pallidum*. Tp0971 was previously reported as a tethered inner membrane protein, which is disputable. To investigate the localization of Tp0971, *T. pallidum* was encapsulated in agarose gel and observed by fluorescence microscopy. Two proteins, FlaA2 and Tp0136, located in the inner flagellum and outer membrane of *T. pallidum,* respectively, served as a position reference (27, 28). As shown in Fig. 3, bright blue fluorescence represents that *T. pallidum* was successfully stained with DAPI. The group of *T. pallidum* with intact outer membranes, the group of *T. pallidum* membranes permeated by Triton X-100, and the group of *T. pallidum* without outer membranes were marked briefly as intact, permeabilized, and removed groups, respectively. In the control experiment, the intact group incubated with FlaA2 antibodies did not show immunofluorescence, while the permeabilized group and removed group showed green fluorescence, which was consistent with the fact that FlaA2 exists in the inner membrane and outer periplasm of *T. pallidum*. Similarly, the intact group and permeabilized group incubated with recombinant Tp0136 antibodies showed green fluorescence, but the removed group did not show green fluorescence, indicating that Tp0136 is an outer membrane protein of *T. pallidum*. In the first row of Fig. 3, the location of Tp0971 is shown. *T. pallidum* cells incubated with recombinant Tp0971 antibodies showed green fluorescence in all groups, indicating that Tp0971 exists in the outer membrane, inner membrane, and periplasm of *T. pallidum*.

### The evaluation of the immune-protective effect of Tp0971

**Fig 4:**
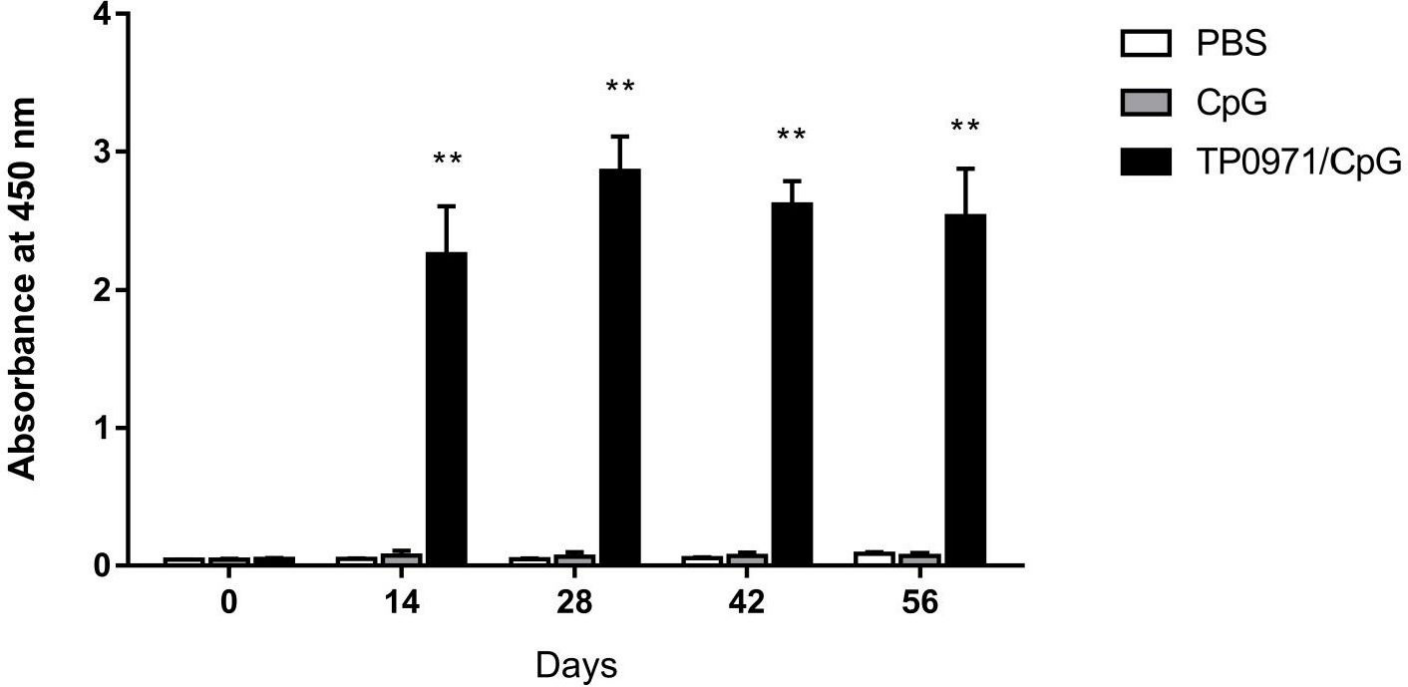
Absorbance values of anti-Tp0971 IgG in the serum of New Zealand rabbits. New Zealand rabbits were immunized with the Tp0971 protein (150 µg) in combination with CpG ODN adjuvants at 0, 14, 28, 42, and 56 days. The serum IgG levels were analyzed by ELISA. The means and standard derivations for the antibody levels were calculated from 69 rabbits (*P < 0. 05; **P < 0. 01).

To evaluate the immune-protective effect of Tp0971, the specific antibody levels in New Zealand rabbits induced by Tp0971/CpG were determined by ELISA. The control groups for the experiment involved New Zealand rabbits injected with phosphate-buffered saline (PBS) alone and CpG adjuvant alone. Following the initial immunization, venous blood was collected from the marginal ear vein of three groups of New Zealand rabbits at 2-week intervals. The collected blood samples were then processed to isolate the serum for the purpose of determining antibody levels using ELISA. The results of ELISA showed that high levels of Tp0971-specific antibodies were detected in the second week after immunization with Tp0971. These antibody levels remained consistently higher compared to the CpG and PBS control groups (Fig. 4). This suggests that Tp0971 has a high level of immunogenicity and is capable of inducing significant humoral immune responses in New Zealand rabbits.

**Fig 5:**
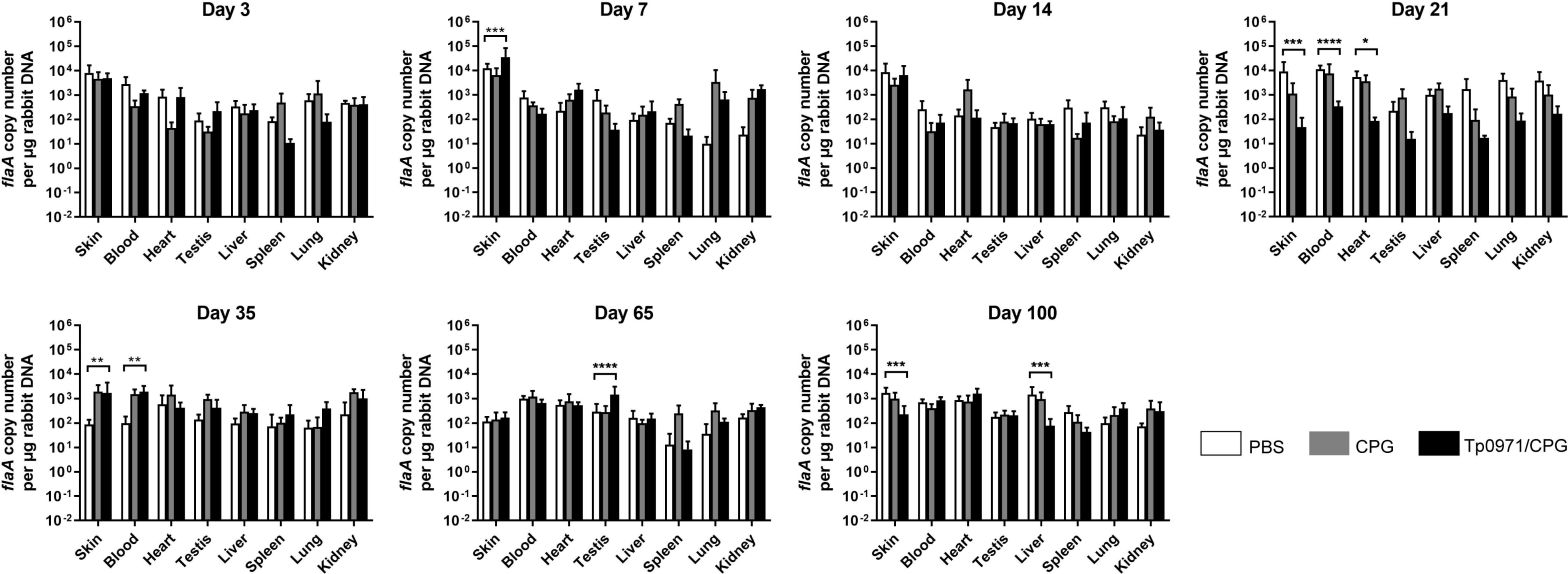
The *T. pallidum* burden in the three groups of New Zealand rabbits at different times after infection. The *flaA* DNA concentrations in the skin, blood, heart, testes, liver, spleen, lung, and kidney of immunized New Zealand rabbits were evaluated by using qRT-PCR after 3, 7, 14, 21, 35, 65, and 100 days of infection. Black, white, and gray represent Tp0971/CpG, PBS, and CpG groups, respectively. Statistical significance is indicated with asterisks (*P < 0.05; **P < 0.01; ***P < 0.001; ****P < 0.0001).

**Fig 6:**
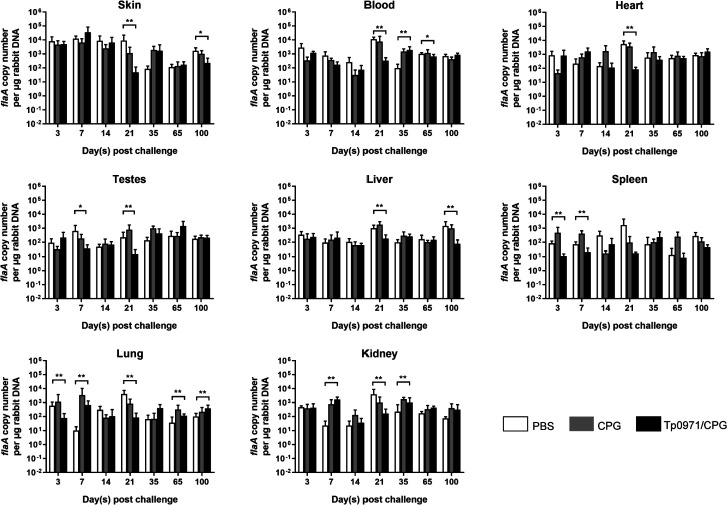
The spread trend in different tissues and blood of *T. pallidum* in New Zealand rabbits. The *T. pallidum* burden was evaluated in different tissues and blood of New Zealand rabbits immunized with Tp0971/CpG, CpG, and PBS using qRT-PCR. The *flaA* DNA copies in the skin, blood, heart, testes, liver, lung, spleen, and kidney were detected after 3, 7, 14, 21, 35, 65, and 100 days of *T. pallidum* infection. Statistical significance is indicated with asterisks (*P < 0.05; **P < 0.01).

In order to verify whether Tp0971/CpG can inhibit the spread of *T. pallidum* to distal organ tissues, we performed qRT-PCR to measure the *T. pallidum* DNA levels in the tissues of three groups of New Zealand rabbits after different infection times. The *T. pallidum* burden in most tissues and at various sites showed no significant difference after infection. The *T. pallidum* burden in the skin of the Tp0971/CpG-immunized group was higher than the PBS- and CpG-immunized groups on the 7th and 35th days. However, on the 21st and 100th days, the *T. pallidum* burden in the skin of both control groups was higher than the Tp0971/CpG-immunized group. On the 21st day after infection, the Tp0971/CpG-immunized group was significantly lower than the control group in the skin, blood, and heart. However, on the 65th day, the *T. pallidum* burden in the testes of both control groups was lower than that in the Tp0971/CpG-immunized group. Interestingly, on the 100th day, the *T. pallidum* burden in the skin and liver showed an opposite trend (Fig. 5). As presented in Fig. 6, the *T. pallidum* burden in the skin, blood, heart, testes, liver, lung, spleen, and kidney of New Zealand rabbits immunized with Tp0971/CpG, CpG, and PBS was analyzed after 3, 7, 14, 21, 35, 65, and 100 days of *T. pallidum* infection. On the 21st day after infection, the *T. pallidum* burden in all samples, except the spleen of New Zealand rabbits immunized with Tp0971/CpG, was significantly lower than control groups. The *T. pallidum* burden in the spleen of the Tp0971/CpG-immunized group was lower than the control group only on the third and seventh days after infection. Interestingly, the *T. pallidum* burden in the lung of the Tp0971/CpG group was significantly lower than the control groups on the 3rd, 7th, and 21st days after infection but was significantly higher on the 65th and 100th days. The *T. pallidum* burden in blood was significantly higher in the Tp0971/CpG-immunized group than that in the control group on the 35th and 65th days. However, the *T. pallidum* burden in the skin, blood, heart, testes, liver, lung, and kidney was significantly lower than that in control groups on the 21st day after infection (Fig. 6). This phenomenon is consistent with the results that the spread of *T. pallidum* in New Zealand rabbits was a cyclic and repeated dynamic process.

### Tp0971 delayed ulcer development in New Zealand rabbits

**Fig 7:**
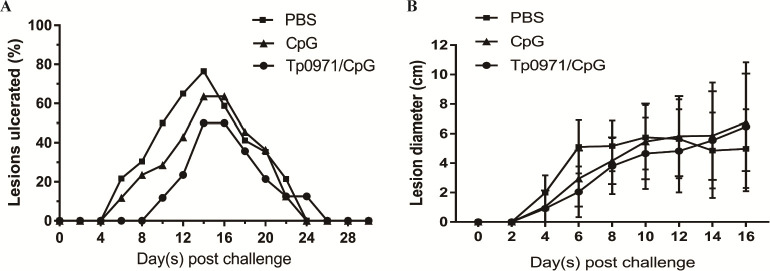
Ulcer development in New Zealand rabbits’ skin. (**A**) The rates of skin ulcers were measured in the three groups on different infection days. (**B**) The induration diameter of lesions in the three groups after *T. pallidum* challenge.

**Fig 8:**
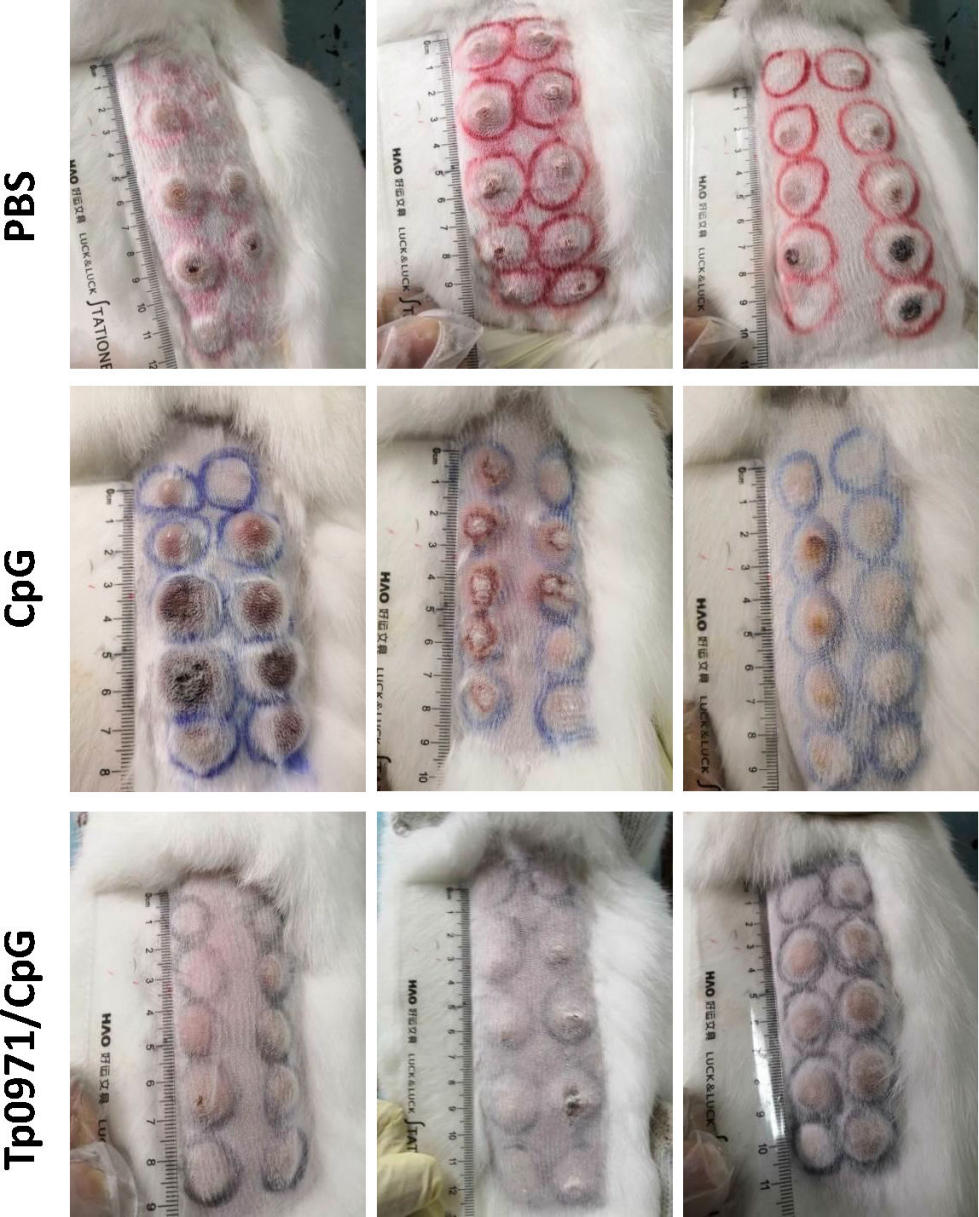
Indurated lesions of injection sites on the back skin of New Zealand rabbits. Indurated lesions from 10 sites on the backs of New Zealand rabbits were observed on the 21st day after *T. pallidum* challenge. The Tp0971/CpG-immunized rabbits had fewer ulcerative lesions than the unimmunized control groups, and the lesion diameter in the control animals was larger than that in immunized animals on the 14th day after infection.

The progression of skin lesions in the *T. pallidum*-infected New Zealand rabbits’ skin was continuously observed. The injection sites on the skin were found to develop skin ulcers (Fig. S1; Fig. 7), similar to the chancre symptom in syphilis patients. Clinically, patients with syphilis often exhibit pathological signs on the skin or mucosa, such as chancre and syphilis rashes during the early stage of infection. Similarly, skin manifestations can also occur in rabbits after intradermal vaccination with *T. pallidum* on the back of New Zealand rabbits. Therefore, investigators commonly use the changes in redness, induration, and ulcers at the inoculation site as a reference to assess the protective effect of the vaccine (29). In the PBS and CpG adjuvant control groups, the inoculation site of New Zealand rabbits presented swollen induration on the sixth and eighth days, and the ulcers began to heal on the 16th or 18th day (Table 1; Fig. S1 and S2). While the skin ulcers in the Tp0971/CpG-immunized group gradually developed on the 10th day and started to heal on the 14th day after *T. pallidum* infection, the healing period in the Tp0971/CpG-immunized group was shorter than that in the control groups (Fig. 7A; Fig. S3). In the Tp0971/CpG-immunized group of New Zealand rabbits, the induration diameter and ulcer rate were smaller compared to the PBS and CpG control groups (Table 1; Fig. 7B and 8). The ulcer rate in the Tp0971/CpG-immunized group was 50%, which was lower than the ulcer rates observed in the PBS (76.47%) and CpG (63.64%) control groups (Table 1). This suggests that Tp0971 may reduce ulcerative lesions, delay the onset of ulcers compared to PBS and CpG controls, and promote the healing of ulcers.

**TABLE 1:** Lesion alteration in New Zealand rabbits after *T. pallidum* infection

	PBS	CpG	Tp0971/CpG
Diameter^*c*^ (cm)	5.75	6.76	5.54
Ulceration^*d*^ (%)	76.47	63.64	50
Induration^*a*^	6	8	10
Heal^*b*^	16	18	14

^*a*^ The earliest appearance of induration.

^*b*^ The time of the start of healing.

^*c*^ Maximum diameter during infection.

^*d*^ Maximum ulcer rate during infection.

### Tp0971 promoted inflammatory cell infiltration after immunization

**Fig 9:**
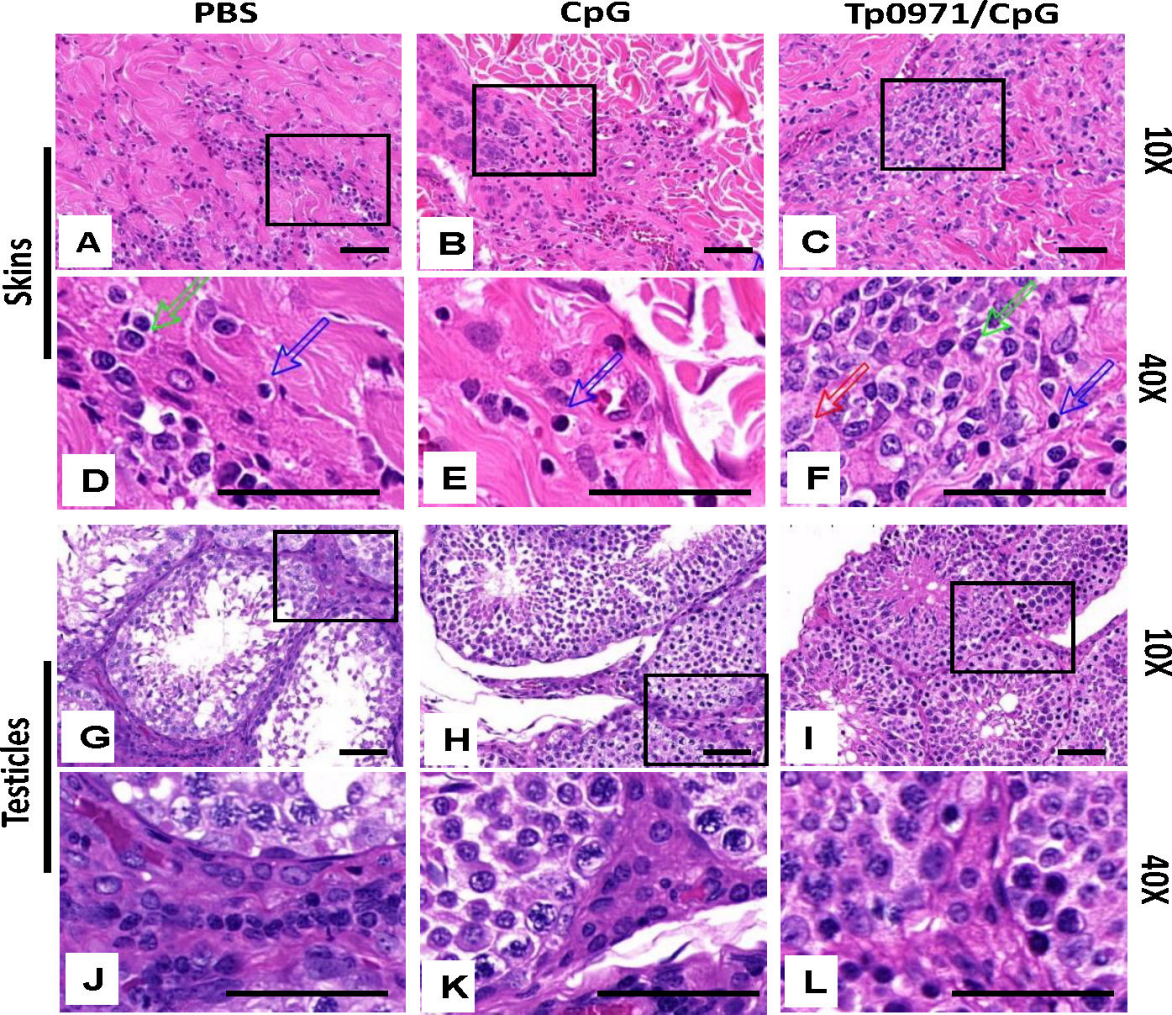
Histopathological alteration in the skin and testicular tissues of New Zealand rabbits after *T. pallidum* infection. (**A to F**) The samples of skin from PBS, CpG, and Tp0971/CpG are presented in panels A, B, C, D, E, and F. Panels D, E, and F are magnifications of the black box in panels A, B, and C, respectively. (**G to L**) Panels G, H, I, J, K, and L represent the testicle samples, with panels J, K, and L being magnifications of panels G, H, and I, respectively. All samples were extracted on the 21st day after *T. pallidum* infection and stained with hematoxylin & eosin (H&E). The histopathological findings reveal predominantly lymphohistiocytic interstitial inflammation, characterized by the presence of lymphocytes, macrophages, and plasma cells (blue arrow: lymphocyte; red arrow: macrophage; green arrow: plasma cell). Scale bars were 50 µm, and the magnification factors of the objective lens are indicated on the right side of each row.

Macrophage-mediated opsonization and phagocytosis are essential for *T. pallidum* clearance (30). We analyzed inflammatory cell infiltration in the infected local and distal testicular tissues using pathological tissue sections. As shown in Fig. 9, all groups of New Zealand rabbits had pronounced infiltration of inflammatory cells (chiefly lymphocytes, macrophages, and plasma cells) at the inoculation site. In the Tp0971/CpG-immunized group, there was noticeably more pronounced inflammatory cell infiltration but fewer inflammatory cells in the testicular tissues (Fig. 9I and L). This may indicate that inflammatory cell infiltration that occurs at the site of infection may contribute to the partial clearance of *T. pallidum* and inhibit its spread to distal tissues.

## DISCUSSION

### The invasion process of *T. pallidum* in New Zealand rabbits

Syphilis, caused by *T. pallidum*, is a chronic bacterial infection disease. It can progress over the years through a series of clinical stages and symptoms and lead to irreversible neurological or cardiovascular complications without treatment (6). It is well known that skin ulcers occur and heal in a circular pattern during the process of *T. pallidum* infection (20). After a long period of asymptomatic infection, untreated patients may progress to stage III syphilis, which involves the development of a multisystem-damaging disease, including damage to the neurological and cardiovascular systems. Some clinical cases have indicated that *T. pallidum* can still be isolated and detected in the cerebrospinal fluid of syphilis patients with serological RPR (The nontreponemal rapid plasma reagin) negative results or without clinical symptoms (31, 32). This suggests that *T. pallidum* infection *in vivo* is a continuous and complex process, and the immune system cannot completely eliminate *T. pallidum* from the body. However, the exact process of its spread *in vivo* remains unclear and difficult to investigate.

We established a *T. pallidum* infection model in New Zealand rabbits and found that *T. pallidum* spread rapidly to multiple tissues and organs of New Zealand rabbits on the third day after *T. pallidum* infection. Furthermore, the *T. pallidum* burden in the blood, heart, lung, and kidney significantly peaked on the 21st day and then decreased. The significance of the *T. pallidum* burden in tissues other than the skin decreased after 21 days, potentially due to the activation of acquired immunity, the organism’s own immune clearance, and the spread of *T. pallidum* to other distal tissues. These findings are consistent with those of Cumberland Cumberland and Turner (33). The spleen had a lower *T. pallidum* burden compared to the skin and blood, which may be attributed to the fact that the spleen is the largest peripheral immune organ and exhibits robust immune clearance during the early stage of infection.

On the third day after infection, *T. pallidum* spread to various tissues. However, the *T. pallidum* burden in the skin was significantly higher compared to the blood and other tissues. This difference became even more pronounced on the seventh day. It means that *T. pallidum* rapidly propagated in the first 2 weeks of infection at the injection sites in the skin and then spread rapidly to other organs after the second week. The disappearance of the significant difference in the *T. pallidum* burden on the 21st and 35th days can be attributed to the immune clearance and bactericidal effect of the body immune system. Interestingly, on the 65th day, the *T. pallidum* burden in blood was significantly higher than that in other tissues and disappeared again on the 100th day. This pattern may be a result of the immune escape mechanisms employed by *T. pallidum,* and *T. pallidum* proliferated in the injection sites on the skin and was released into the blood once again. This ongoing battle between *T. pallidum* and the host immune system results in long and chronic infection. Overall, the *T. pallidum* burden in the tissues of New Zealand rabbits follows a dynamic balance of repeatedly decreasing and increasing.

### The localization of Tp0971

The outer membrane is a durable and adaptable permeability barrier that enables Gram-negative bacteria to thrive in unpredictable and hostile environments as free-living organisms, pathogens, or both (2). The *T. pallidum* outer membrane must support homeostatic, protective, and virulence-related functions, and *T. pallidum* as a bacterium with a markedly limited biosynthetic capacity and an exclusively pathogenic, invasive lifestyle is capable of causing protracted, and even lifelong, infection in humans (2, 8, 11). In 1980, when the phase partitioning technology of Triton X-114 was applied to the separation of membrane proteins, the localization of membrane proteins in many bacteria was investigated (34), including the localization of hypothetical membrane proteins and antigen hydrophobicity of *T. pallidum* (35, 36). Additionally, the signal peptides of highly immunogenic membrane proteins containing lipid-modified motifs were confirmed through DNA sequencing technology (37 – 40). This suggests that the hydrophobic properties and membrane binding of natural lipoproteins are related to their lipid components. Subsequently, immunoelectron microscopy and immunofluorescence assay were employed to determine the membrane localization of *T. pallidum* lipoproteins. The use of gel microdroplets is a novel strategy for investigating the molecular architecture of treponemal organisms (41, 42). This method allows the integrity of the outer membrane’s integrality of *T. pallidum* to be maintained during the experimental process (41). The Tp92 (Tp0326), Tp47 (Tp0574), and Tp0136 membrane proteins of *T. pallidum* had been located successfully by gel microdroplets (28, 42, 43). In our previous animal experiments, we found that the production of Tp0971 antibodies is highly induced in rabbits infected with live *T. pallidum* but not inactivated *T. pallidum*. This finding is consistent with the characteristics of an infection-dependent antigen (20). It has been reported that the Tp0971 gene product of *T. pallidum* (Tp0971) is a periplasmic lipoprotein believed to be tethered to the inner membrane of this organism (14, 15). We applied gel microdroplets to investigate the location of Tp0971 in *T. pallidum*. Our results showed that Tp0971 exists in the outer membrane, inner membrane, and periplasm of *T. pallidum*, indicating that Tp0971 might be a membrane lipoprotein protein that exists in the inner and outer membranes of *T. pallidum*. This finding is consistent with the infection-dependent antigen properties (20). On the other side, it has been reported that Tp0971 is capable of binding mammalian iron transport proteins, as well as binding zinc and the iron-sequestering lactoferrin for iron acquisition. This suggests that Tp0971 might be exposed on the outer membrane of *T. pallidum*, allowing it to have access to these nutrients within human body fluids (14). This characteristic is consistent with our results. Numerous reports have demonstrated that infection-dependent antigens are crucial for pathogenic infections and represent new options for diagnostic markers or vaccine development (21 – 23).

### The immune-protective function of Tp0971

Syphilis continues to be a major public health problem worldwide. Recent increases in the number of syphilis cases, combined with the lack of an efficient vaccine against *T. pallidum* for humans, highlight an urgent need for the design and development of an efficacious syphilis vaccine (44). Syphilis elimination will require the development of an effective vaccine that has thus far remained elusive (45). An effective syphilis vaccine should elicit antibodies to *T. pallidum* surface antigens to induce pathogen clearance through opsonophagocytosis. Although the combination of bioinformatics, structural, and functional analyses of *T. pallidum* genes to identify putative outer membrane proteins resulted in a list of potential vaccine candidates, there is still little knowledge about the transcriptional regulation of these genes during infection. This knowledge gap presents a limitation to vaccine design and development. Immunity generated against an antigen that can be downregulated or even silenced at the transcriptional level without affecting virulence would not induce clearance of the pathogen, thus allowing disease progression (46).

First, *T. pallidum* invades the organism through damaged skin or mucosa. Subsequently, the TLRs or pattern recognition receptors of monocytes/macrophages and dendritic cells are activated (3, 4). *T. pallidum* lipoproteins are mostly located under the outer membrane; pattern recognition molecules under the outer membrane of spirochetes do not easily bind to TLRs or other pattern recognition receptors on monocytes or dendritic cells; therefore, *T. pallidum* does not easily activate the innate immune recognition system after invasion. In addition, the outer membrane structure of *T. pallidum* is unique and has some low density, instability, and mutability of outer membrane proteins, which induce the production of limited *T. pallidum* antibodies (e.g., antibodies against membrane proteins such as TpN17, TpN37, and TpN47) (47). Based on our results, Tp0971 might be a membrane lipoprotein that exists in the inner and outer membranes, and our previous study indicated that Tp0971 reacts with syphilis patients’ serum with high sensitivity and specificity (20). Meanwhile, the Tp0971 antibody can be highly produced in rabbits infected only by live *T. pallidum* but not inactivated *T. pallidum*, which is consistent with the characteristics of infection-dependent antigens (20). We selected Tp0971 as the immunogen for immunization of New Zealand rabbits and found that Tp0971 induced the production of high levels of specific IgG antibodies. Interestingly, the *T. pallidum* burden in all samples except the spleen of New Zealand rabbits immunized with Tp0971/CpG was significantly lower than control groups on the 21st day after infection. This reduction in the burden might result from immune clearance mediated by Tp0971/CpG antibodies and the bactericidal effect of the body immune system. Combined with the previous results, which showed that *T. pallidum* spread rapidly to other organs in the first 2 weeks and reached a significant maximum quantity on the 21st and 35th days, we can conclude that Tp0971/CpG has the ability to activate the human immune system and promote the clearance of *T. pallidum*.

Meanwhile, the induration diameter and ulcer rate of New Zealand rabbits in the Tp0971/CpG-immunized group were lower than those in the PBS and CpG control groups. We also analyzed inflammatory cell infiltration in the infected sites and distal testicular tissues using pathological tissue sections. The Tp0971/CpG-immunized group had more pronounced inflammatory cell infiltration, but fewer inflammatory cells were observed in the testicular tissues. Macrophage-mediated opsonization and phagocytosis are essential for *T. pallidum* clearance (30). This may indicate that inflammatory cell infiltration occurring at the site of infection can partially clear *T. pallidum* and reduce its spread to distal tissues. Additionally, we observed that Tp0971 may reduce the occurrence of ulcerative lesions and delay the onset compared to the PBS and CpG controls, promoting the healing of ulcers. Therefore, it can be concluded that Tp0971/CpG induced the production of high levels of specific IgG antibodies and delayed the development of ulcers. This result is consistent with Tp0751 of *T. pallidum*; New Zealand rabbits immunized with the adhesin protein Tp0751 (45) and the flagellin FlaB (48) showed some resistance to *T. pallidum* infection when testicular inflammation was strong, but it could not completely prevent *T. pallidum* infection.

We speculate that when *T. pallidum* replicates and proliferates after evading local immune clearance of the organism, Tp0971-specific antibodies and inflammatory cells are induced to play certain antibacterial effects, thereby significantly reducing the *T. pallidum* burden on the 21st day of infection. While syphilis infection is accomplished through the collaboration of multiple *T. pallidum* proteins or molecules, the role of Tp0971 in the entire process is limited and does not achieve a complete and continuous protective effect. Moreover, *T. pallidum* can produce some temporally expressed proteins or other small molecules, such as vesicles and microRNA, to mediate the interaction between the pathogen and the organism and assist in the survival of the pathogen in the host. Furthermore, *T. pallidum* can generate different antigenic variation responses to evade clearance, causing spirochetes to grow again in the late stage of infection.

### Conclusion

Syphilis, which is caused by the sexually transmitted bacterium *T. pallidum* subsp. *pallidum*, has an estimated 6.3 million cases worldwide per year, but the evolution and epidemiology of the epidemic are poorly understood (49). The well-recognized capacity of the syphilis spirochete for early dissemination and immune evasion has earned the designation of “the stealth pathogen.” Despite the many hurdles to studying syphilis pathogenesis, most notably the difficulty of culturing and genetically manipulating *T. pallidum* (8), New Zealand rabbits infected with *T. pallidum* displayed similar symptoms to clinical patients. *T. pallidum* rapidly propagates in the first week of infection at the injection sites on the skin and then spreads rapidly to other organs within the first 2 weeks. After the host immune system is activated, *T. pallidum* employs immune escape mechanisms to evade immune clearance, leading to a long and chronic infection. Therefore, the burden of *T. pallidum* in various organs fluctuated between decreasing and increasing throughout the entire infection process. Tp0971/CpG-immunized New Zealand rabbits were unable to completely resist *T. pallidum* infection or inhibit the spread of *T. pallidum* in the New Zealand rabbit body. However, Tp0971/CpG immunization did induce high levels of Tp0971-specific antibodies, which helped in delaying skin damage and promoting healing at the infected site of *T. pallidum* in New Zealand rabbits. While New Zealand rabbits have interspecies differences from humans and the experimental procedure cannot fully replicate the actual replication of *T. pallidum* in humans, it still offers valuable insights into the mechanism of continuous infection and spread of *T. pallidum* in the host. Further evaluation is required to understand the role of the infection-dependent antigen Tp0971 as an immunogen in the proliferation and spread of *T. pallidum. T. pallidum* infection in humans is a complex and long process, and there is a continuous need for exploration into its specific pathogenic mechanism, pathogenesis, and vaccine research.

## MATERIALS AND METHODS

### Testicular inoculation

We selected New Zealand rabbits weighing about 2.5–3 kg for testicular inoculation and conducted RPR and TPPA (*T. pallidum* particle agglutination) tests, which yielded negative results. To ensure equanimity, we injected 3 mg/kg acepromazine into the muscle of New Zealand rabbits. The scrotal area was thoroughly sterilized with iodophor. The frozen *T. pallidum* Nichols strain was then transferred from a −80°C refrigerator to an ice box for temporary storage. After the complete thawing of the frozen *T. pallidum*, 500 µL of *T*. *pallidum* suspension (1 × 10^7^ /mL, Nichols strain) was injected into three different points of the testicle. Following the injection, the rabbits were fed with antibiotic-free rabbit forage twice a day at a constant temperature (18°C–20°C) and constant humidity (55%–65%). After 15 days of inoculation, when obvious orchitis occurred, 2 mL of blood was drawn from the margin ear vein and transferred to a microcentrifuge tube. The tube was then centrifuged at 3,000 × *g* for 10 minutes to separate serum, followed by 30 minutes of incubation at 37°C. If the RPR and TPPA serological tests of the blood yielded positive results, the animal model of syphilis infection in the testes was successfully established.

### The purification and preparation of *T. pallidum*

The serum of New Zealand rabbits, inoculated with *T. pallidum*, was tested using RPR and TPPA. When both results were positive, the serum titer of RPR was found to be more than 1:4. Following this, the New Zealand rabbits were secured on the rabbit restraint plate and euthanized through intravenous injection of 90 mg/kg pentobarbital or air embolization for euthanization. The scrotum was moisturized and sterilized with 70% ethanol. The parenchyma testis was separated and washed with normal saline in a sterile dish. The testicular parenchyma was transferred into an Eppendorf tube with 25 mL of normal saline and vibrated for 45 minutes. After the vibration, the supernatant was centrifuged at 500 × *g* for 8 minutes. The resulting supernatant was then transferred into a new sterile Eppendorf tube and centrifuged again at 12,000 × *g* for 10 minutes. After centrifugation, the pellet was resuspended in 1 mL of aseptic normal saline. The motility of *T. pallidum* was observed by a dark field microscope. The cell density was adjusted to 1 × 10^7^ /mL for intradermal inoculation.

### Intradermal inoculation of *T. pallidum*

New Zealand rabbits, weighing 2.5–3 kg, were administered 3 mg/kg of acepromazine via intramuscular injection for stabilization. Ten inoculation points, each with a diameter of 4 cm, were shaved and marked on the skin of the New Zealand rabbits. Each inoculation point was sterilized with iodine and injected with 0.1 mL of *T. pallidum* cell suspension (1 × 10^7^ /mL). The skin alterations of New Zealand rabbits were observed and recorded. Additionally, blood samples were obtained from the auricular vein of New Zealand rabbits every 2 days. The antibody titer was evaluated using RPR and TPPA tests. When both results were positive and indurated lesions appeared, it indicated that the animal infection model had been established successfully.

### The spread evaluation of *T. pallidum* in New Zealand rabbits

Sixty-nine male New Zealand rabbits (3.0–3.5 kg, 13–15 weeks of age), obtained from the animal department of the University of South China and with negative VDRL and FTA-Abs serology, were used. Fourteen days after the last immunization, 10 spots on the back skin of New Zealand rabbits were shaved and intradermally injected with 0.1 mL of *T. pallidum* (amount: 1 × 10^7^, Nichols strain) in 0.9% saline. Rabbits were monitored daily for erythema, induration, and ulcerative lesions, and lesion diameters were measured every 2 days to evaluate the protective effects. Three rabbits in each group were randomly euthanized at 3, 7, 14, 21, 35, 65, and 100 days after *T. pallidum* challenge. Blood and tissues were harvested for examination using hematoxylin & eosin staining and qRT-PCR.

### DNA extraction and purification

The DNA of blood, lesion skin, and tissue samples was extracted using a QIAamp DNA Mini Kit (Qiagen, Shanghai, China) according to the manufacturer’s instructions. A total of 180 mL of lysis buffer (containing 10 mM Tris, pH 8.0, 0.1 M EDTA, and 0.5% SDS) was mixed with 25 mg of tissue (10 mg for spleen and 200 µL for blood), and the mixture was homogenized using the Qiagen TissueLyser LT operating at 30 Hz for 40 seconds. Proteinase K (5 mg) was added, followed by digestion at 56°C for 3 hours. Then, the samples were treated with RNase A (400 µg) and incubated at 70°C for 10 minutes. For reproducibility, three tissue samples from each rabbit organ were analyzed. The tissue extracts were eluted in 100 µL of Gibco PCR Grade Distilled Water (Thermo Fisher Scientific). The DNA from samples was stored at −80°C until analysis by qRT-PCR. The DNA concentration was measured using a UV/VIS spectrophotometer (Beckman Coulter Canada, Mississauga, ON, Canada).

### Quantitative real-time PCR

qRT-PCR was performed using DNA extracted from the blood and tissues of *T. pallidum*-challenged New Zealand rabbits according to the manufacturer’s instructions. The primers used for the *T. pallidum flaA* (endoflagellar sheath protein, GenBank number M63142) and New Zealand rabbit MMP-1 (collagenase-1 precursor, GenBank accession number AH005676) genes were as previously described (45). The qRT-PCRs were carried out in a 20 µL volume. All assays were run on a LightCycler 96 apparatus (Roche, Basel, Switzerland). The LightCycler 96 Application Software uses predefined dye-specific fluorescence threshold values to calculate the Cq value of a sample. The predefined fluorescence threshold value used in an experiment depends on the specified detection format (dye). The Cq value for a sample is only displayed in the results if the sample is determined “positive” by the positive/negative filter algorithm or by the gene-specific threshold set manually by the operator. The Cq threshold and the positive/negative threshold are two independent thresholds that are not correlated. Cq values are calculated once for each sample and do not change when the positive/negative threshold is changed manually by the operator. The Cq threshold cannot be changed by the operator. In this experiment, SYBR Green I and ResoLight dye were employed, with a predefined fluorescence threshold value set at 0.2. The qRT-PCR conditions for amplification of *flaA* and MMP-1 were as follows: preincubation at 95°C for 10 minutes; amplification for 40 cycles at 95°C for 15 seconds, 55°C for 20 seconds, and 72°C for 20 seconds; and melting curve analysis for one cycle at 95°C for 10 seconds, 65°C for 60 seconds, and 97°C for 1 second.

### Gel microdroplet method

According to Luthra et al.’s experiment (42), gel microdroplets were used to detect *T. pallidum* lipoproteins. The *T. pallidum* with intact outer membrane group, the *T. pallidum* membrane permeated by Triton X-100 group, and the *T. pallidum* without outer membrane group were labeled as intact, permeabilized, and removed, respectively. Cell suspension of the intact and permeabilized groups was prepared using the methods of purification and preparation of *T. pallidum*. In the removed group, the experimental method was slightly modified. After *T. pallidum* was resuspended in normal saline, citrate solution was added to the purified and concentrated *T. pallidum* suspension, and it was shaken repeatedly to mix. This process was repeated three times. Then, the pellet was resuspended in normal saline. Sodium citrate was employed to remove the outer membrane of *T. pallidum* (50). *T. pallidum* treated with sodium citrate was encapsulated in gel microdroplets (Sigma-Aldrich). In the intact and removed groups, the encapsulated *T. pallidum* was then transferred to 500 µL of normal saline containing 10% bovine serum. In the permeabilized group, a normal saline solution containing 0.2% Triton X-100 and 10% bovine serum was used for permeabilizing the outer membrane. The intact cell group did not contain 0.2% Triton X-100. Three groups were incubated at 4°C overnight. The antibody for periplasmic flagellar sheath protein (FlaA2), outer membrane protein (rTp0136), and Tp0971 was diluted 1:100-fold. *T. pallidum* was then incubated with these antibodies at 4°C overnight and washed five times with saline. For labeling, 400 µL of Alexa Fluor 488 (green fluorescence)-labeled anti-mouse IgG (2 µg/µL, Abcam) was used. A bright blue fluorescence reagent (1 µg/µL, Solebaol) was used for incubation with *T. pallidum* at 4°C for 8 hours. The cells were washed five times afterward. Finally, the agarose with *T. pallidum* was observed under a fluorescent inverted microscope.

### Recombinant protein expression and purification

The plasmid pet32a^+^/Tp0971 was constructed for the expression in *Escherichia coli* BL21 by Sangon Biotech, Shanghai, China. Expression and purification of the Tp0971 protein were performed according to a previous description (20). The concentrations of the purified recombinant proteins were estimated using a BCA protein assay kit (Pierce, Rockford, IL, USA). A polymyxin B solution with a concentration of 100 mg/mL was prepared and mixed with rTp0971 at a ratio of 1,000:1 for the elimination of endotoxin. An endotoxin test kit was applied to determine the concentration of protein endotoxin by the quantitative colorimetric matrix method.

### Rabbit immunization

Sixty-nine male New Zealand rabbits, weighing between 3.0 and 3.5 kg and aged between 13 and 15 weeks, were obtained from the animal department of the University of South China and tested negative for VDRL and FTA-Abs serology. The rabbits were randomly divided into three groups. In the Tp0971/CpG immune group, New Zealand rabbits were injected with a mixture of 0.5 mL containing 150 µg of Tp0971 and 50 µg CpG. The injection was administered at the quadriceps femoris on both sides using 0.4 mL of the Tp0971/CpG mixture, and 0.1 mL of the mixture was subcutaneously injected at the shoulder. In addition to the Tp0971/CpG immune group, there were two other control groups in the experiment. The New Zealand rabbits injected with 50 µg CpG adjuvant alone constituted the CpG control group. The PBS blank control group received injections of 0.5 mL phosphate-buffered saline. All the New Zealand rabbits, regardless of their group, were immunized four times, with each immunization occurring once every 2 weeks.

### *T. pallidum* challenge

Fourteen days after the last immunization, 10 spots on the back skin of New Zealand rabbits were shaved and intradermally injected with 0.1 mL of *T*. *pallidum* cells (amount: 1 × 10^7^, Nichols strain) in 0.9% saline administered. Rabbits were monitored daily for erythema, induration, and ulcerative lesions, and lesion diameters were measured every 2 days to evaluate the protective effects. At 3, 7, 14, 21, 35, 65, and 100 days after *T. pallidum* challenge, three rabbits in each group were randomly euthanized, and blood and tissues were harvested for examination by hematoxylin & eosin staining and qRT-PCR.

### Evaluation of humoral immune responses by ELISA

Blood samples were collected from the auricular vein of New Zealand rabbits every 2 weeks, and the antibody titer was evaluated using indirect ELISA. Recombinant Tp0971 was diluted to a concentration of 5 µg/mL. First, 96-well polystyrene plates (Costar, Corning, NY, USA) were washed with PBST two times and then coated with Tp0971 antigens at 4°C overnight. The plates were then blocked with PBST solution containing 5% fat-free milk at 37°C for 2.5 hours. New Zealand rabbits’ sera were diluted in PBST solution using a doubling dilution series and added to the plates, which were incubated at 37°C for 2 hours. The plates were washed six times with PBST again. Horseradish peroxidase-conjugated secondary goat anti-rabbit IgG, diluted with PBST solution (1:10,000), was incubated at 37°C for 1 hour. After washing the plates four times with PBST, the absorbance was measured at 540 nm using BioTek Instruments (Winooski, VT, USA). Each experiment was repeated three times.

### Histological analysis

Skin and testicular tissue specimens from New Zealand rabbits were fixed in formalin, sectioned, and subjected to hematoxylin & eosin staining. The stained sections were then observed under a light microscope.

### Statistical analysis

The results are reported as mean ± standard error (SE). The statistical tests were conducted using two-way ANOVA. The analyses were performed using GraphPad Prism 8.0 software (GraphPad Software). A significance level of *P* < 0.05 was considered statistically significant.

## Data Availability

The data sets generated during and/or analyzed during the current study are available from the corresponding author upon reasonable request.

## SUPPLEMENTAL MATERIAL

Supplemental figures spectrum.00047-23-s0001.docx Fig. S1 to S3.

ASM does not own the copyrights to Supplemental Material that may be linked to, or accessed through, an article. The authors have granted ASM a non-exclusive, world-wide license to publish the Supplemental Material files. Please contact the corresponding author directly for reuse.
