# Supplementary material for: Insight into the invasion process and immune-protective evaluation of Tp0971, a membrane lipoprotein from Treponema pallidum
Source: Microbiol Spectr. 2023 Oct 19;11(6):e00047-23. doi: 10.1128/spectrum.00047-23 (PMC10714829; doi:10.1128/spectrum.00047-23)
Supplement: Supplemental figures — Fig. S1 to S3. [file spectrum.00047-23-s0001.docx]

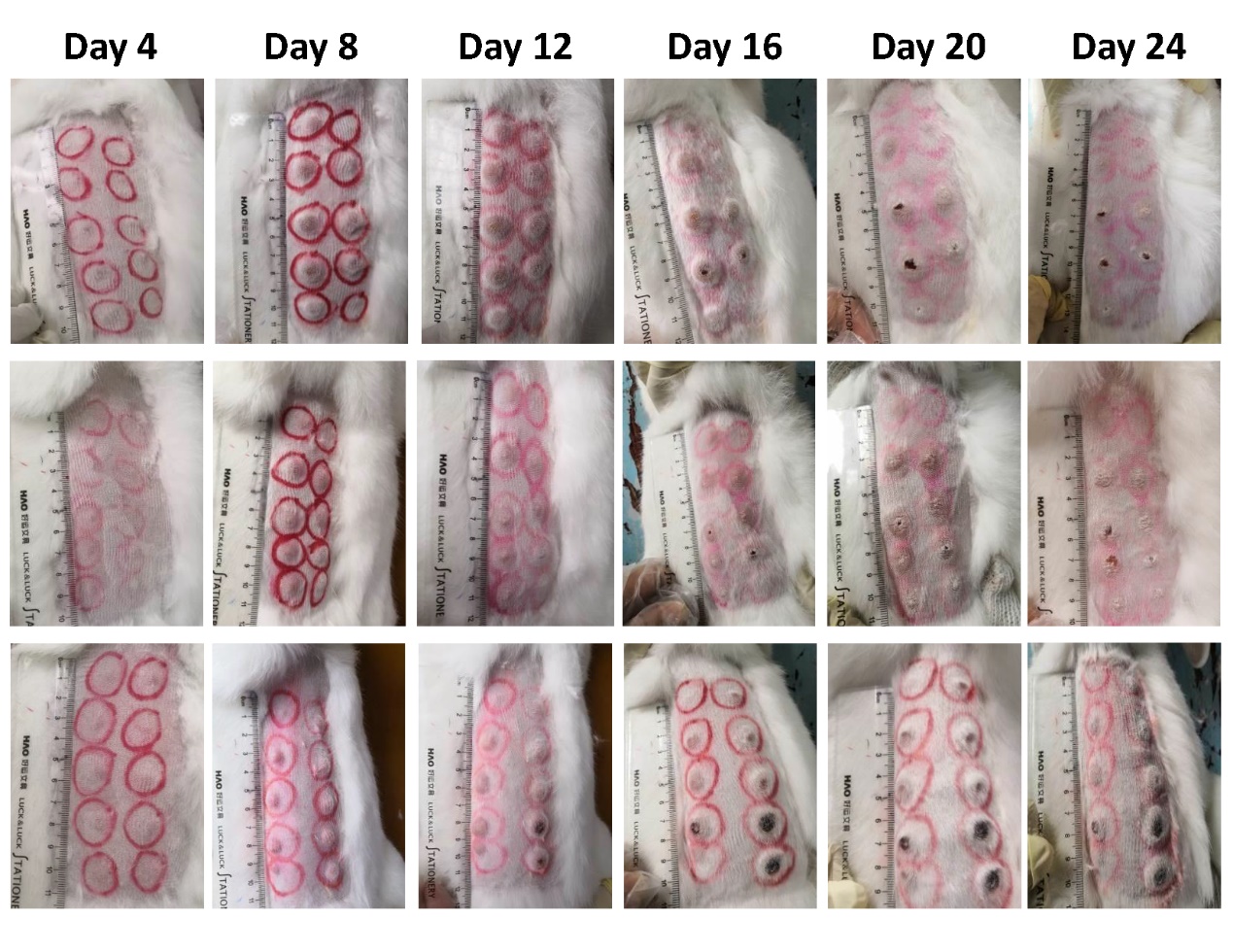


**Fig S1. The process of ulcer alterations at the skin inoculation site of New Zealand rabbit of PBS group.** Three typical New Zealand rabbits were randomly selected from PBS control group, and the development of skin ulcers was recorded at 4, 8, 12, 16, 20 and 24 days after intradermal challenged with *T. pallidum*.


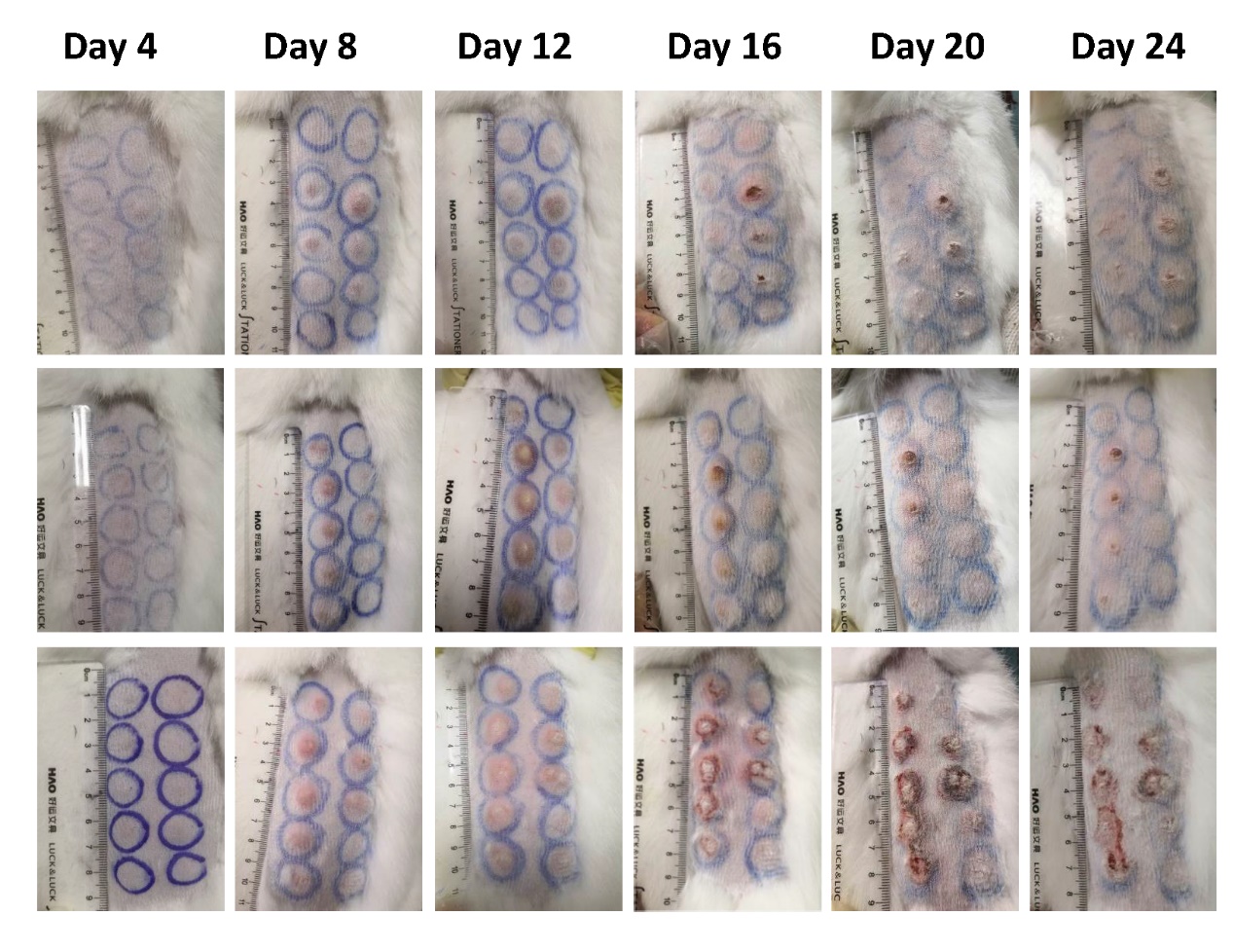


**Fig S2. The progression of ulcer changes at the skin inoculation site of New Zealand rabbits of CpG group.** Three New Zealand rabbits were randomly chosen from CpG control group, and the development of skin ulcers was documented at specific time points: 4, 8, 12, 16, 20 and 24 days after intradermal challenged with *T. pallidum*.


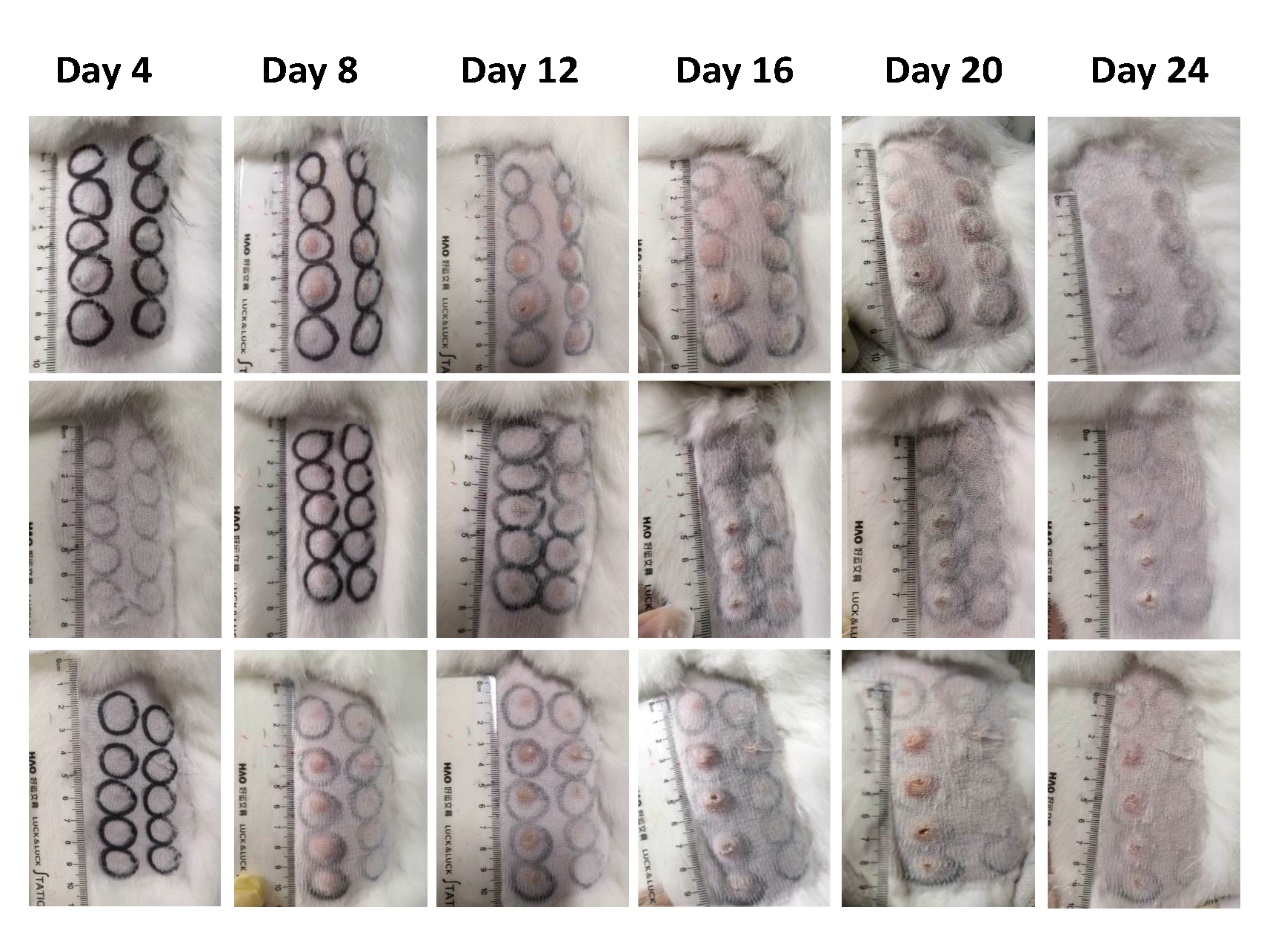


**Fig S3. The progression of ulcer changes at the skin inoculation site of New Zealand rabbits of Tp0971/CpG group.** Three rabbits were randomly selected from Tp0971/CpG Immunized group, and the development of skin ulcers was documented at 4, 8, 12, 16, 20 and 24 days after intradermal challenged with *T. pallidum*.
